# Genome annotations matter: characterizing Ensembl hg38 annotations from 2014 to 2023

**DOI:** 10.1186/s12864-025-12167-8

**Published:** 2025-12-05

**Authors:** Madeline L. Page, Mark E. Wadsworth, Bernardo Aguzzoli Heberle, David W. Fardo, Mark T. W. Ebbert

**Affiliations:** 1 Sanders-Brown Center on Aging, University of Kentucky, Lexington, KY USA; 2 Division of Biomedical Informatics, Department of Internal Medicine, College of Medicine, University of Kentucky, Lexington, KY USA; 3 Department of Neuroscience, College of Medicine, University of Kentucky, Lexington, KY USA; 4 Department of Biostatistics, College of Public Health, University of Kentucky, Lexington, KY USA

**Keywords:** Genome annotations, RNA isoforms, Long-reads, Nanopore sequencing, GTEx

## Abstract

**Background:**

An accurate genome annotation is essential in many contexts, including RNA sequencing studies. Annotations include known genes and isoforms, detailing their location (chromosome, start, and end) and coding sequence, among other important metadata.

**Results:**

We characterized changes in human Ensembl annotations from 2014 to 2023 and the important gains in our biological understanding in recent years. While generally gene and isoform annotations increased (2014: 58,812 genes ; 2023: 62,710), some years dropped (e.g., 2016). A similar pattern exists for the gene and isoform biotypes; both 2015 (19,825) and 2017 (19,828) have fewer genes annotated as protein-coding than 2014 (19,953) and 2016 (19,961)— 2023 has the most (20,048). *PCBP1-AS1* had the most annotated isoforms (296). We quantified expression for isoforms that were new between 2019 and 2023 across nine GTEx tissues (58 samples) to demonstrate our significant gains in understanding recently. We saw 2,054 of these ‘new’ isoforms expressed in cerebellar hemisphere (594 in liver). For many genes, we saw that the relative expression of the ‘new’ isoforms was much greater than the previously known isoforms.

**Conclusions:**

This study demonstrates the importance of an accurate genome annotation to truly understand the underlying complexity of biology that is often oversimplified by ignoring transcriptional complexity.

**Supplementary Information:**

The online version contains supplementary material available at 10.1186/s12864-025-12167-8.

## Introduction

A genome’s annotations are critical in many applications, including RNA sequencing (RNA-seq). Annotations define genomic coordinates for genes, gene features, and isoforms, and provide a framework to define and study individual genes, among other purposes. As new information emerges, annotations undergo regular updates, requiring extensive effort to compile, validate, and curate annotations. Different consortia take distinct approaches to annotation, leading to discrepancies in gene positions and features. As a result, gene annotations remain an evolving challenge.

Since genome annotations are necessary for quantifying gene expression, their accuracy is essential. Recent studies highlighted substantial variation in RNA-seq results depending on annotation source. Specifically, Zhao and Zhang [1] found that only about 16% of genes had identical counts when comparing Ensembl, RefSeq, and the UCSC annotations. Similarly, McCarthy et al. [2] reported only 44% agreement between RefSeq and Ensembl in loss-of-function variant identification. These findings underscore the importance of accurate gene annotations to help researchers accurately quantify gene expression. A critical next step in our understanding throughout biological research, however, is characterizing individual RNA isoforms and their individual functions. Long-read sequencing now enables improved isoform discovery and quantification, and is providing a more complete picture of the human genome [3–5].

Here, we characterize the evolution of human Ensembl gene and isoform annotations over ten years (2014 to 2023), quantifying how annotations changed over time. We also leverage long-read data from 58 GTEx samples, as published by Glinos et al. [4], to quantify isoform expression for those discovered between 2019 and 2023, and describe insights gained from those additional RNA isoforms by assessing their expression across nine tissue types. These data demonstrate the importance of using the most accurate annotation possible.

## Results

### Gene and RNA isoform annotation comparison from 2014 to 2023 reveals a general increase in gene body and RNA isoform discoveries and many annotated isoforms per gene

#### Gene body annotations

**Fig. 1:**
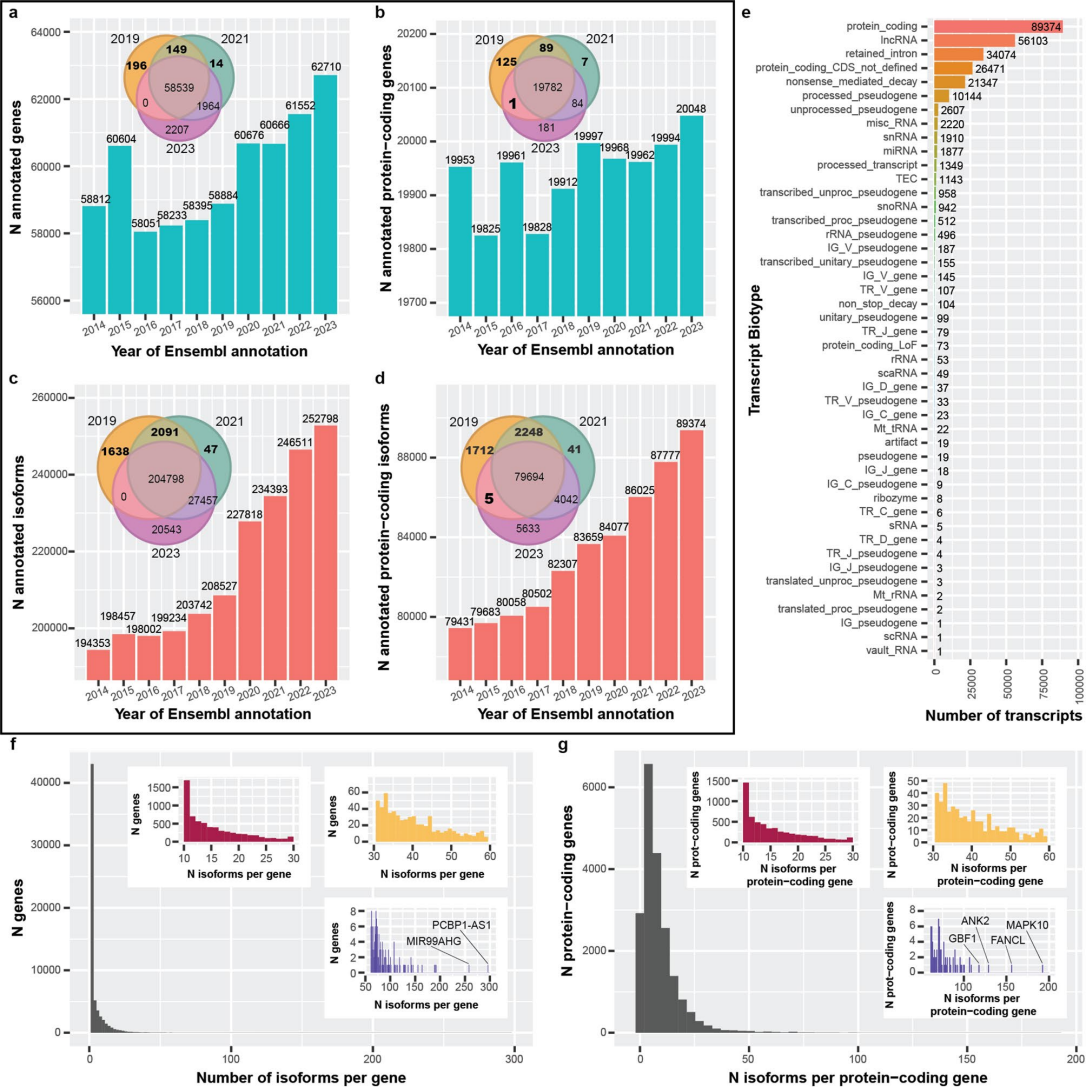
*Gene and isoform annotations generally increased between 2014 & 2023 and reveal complex isoform diversity*. For figures (**a**-**d**), a representative Ensembl annotation was chosen for each year from 2014–2023. **a** The number of annotated genes per year from 2014–2023. After 2016, the overall trend is an increase, with a reasonably sharp increase around 2020. We see that some annotations appear to be dropped completely between years (Venn diagram). **b** Same as (**a**) but restricted to protein-coding genes. In contrast, there were more fluctuations in the number of annotated protein-coding genes, though still a positive trend. One gene was annotated as protein-coding in the 2019 and 2023 annotations but was not in 2021. **c** Looking specifically at RNA isoform annotations, the trend is positive, with a sharp increase in 2020. **d** Similar patterns exist for RNA isoforms annotated as protein-coding. There are five isoforms that were not labeled as protein-coding in 2021 but were in 2019 and 2023. **e** Bar plot showing transcript biotype for all isoforms in 2023. As expected, protein-coding was the most common biotype. Interestingly, retained intron was 3rd, and nonsense-mediated decay was 5th. **f** Histogram showing number of annotated isoforms per gene. Colored, zoomed subplots are shown for convenience. The majority of gene bodies had only one annotated isoform (median = 1; 75th percentile: 4; 85th : 8; 95th : 16), but some had more than 100. The most annotated isoforms for a single gene body was 296 (*PCBP1-AS1*; ENSG00000179818). **g** Similar to (**f**) but showing the number of isoforms per protein-coding gene. The median number of annotated isoforms is 6 (75th percentile: 11 isoforms). The most annotations for a single protein-coding gene body was 192 (*MAPK10*; ENSG00000109339)

To quantify the rate of gene body and RNA isoform discoveries, we compared Ensembl annotations spanning 2014 to 2023 at both the gene and RNA isoform levels (Fig. 1; Supplemental Table S1). Starting with Ensembl v76 (released August 2014), there were 58,764 gene annotations present, compared to 62,710 by Ensembl v109 (released February 2023; Fig. 1a), resulting in an increase of 3,946 (6.7%). We observed a sharp increase in new gene body annotations in 2015 (+ 1,792), followed by a sharper decrease in 2016 (−2,505). Between 2016 and 2023, there was a general increase in gene annotations, including large increases in 2020 and 2023. Comparing gene body annotations between 2019, 2021, and 2023, 196 and 14 were unique to 2019 and 2021, respectively, with 149 shared between them that were missing in 2023 (Venn diagram in Fig. 1a), indicating that many gene body annotations were dropped or their Ensembl ID was changed between 2021 and 2023.

In contrast to the full set of gene body annotations, there were multiple increases and decreases for protein-coding genes (genes where gene_biotype = protein_coding) from 2014 to 2017 (Fig. 1b) and small decreases between 2019 and 2021. Comparing protein-coding gene body annotations between 2019, 2021, and 2023, 125 and 7 were unique to 2019 and 2021, respectively, with 89 shared between them that were not present in 2023 (Venn diagram in Fig. 1b). For interest, there was a single protein-coding gene body annotation present in 2019 and 2023 that was absent in 2021 (Supplemental Table S2); the annotation was present in all three releases, but the gene’s biotype changed from “protein-coding” to “transcribed unprocessed pseudogene” in February 2021 (Ensembl release 103) [6], but reverted to “protein-coding” by December 2021 (Ensembl release 105) [7].

#### RNA isoform annotations

In many instances, a reference genome is only as good as its annotations, especially when it comes to RNA sequencing; thus, while having properly annotated gene bodies is critical, having fully characterized and annotated RNA isoforms for all genes is equally important for understanding an organism’s full complexity. Exactly 194,305 RNA isoform annotations existed in 2014 (Ensembl v76), compared to 252,798 in 2023 (58,493 increase); 19,291 were newly annotated between 2019 and 2020 alone (Fig. 1c). Comparing RNA isoform annotations between 2019, 2021, and 2023; 1,638 and 47 were unique to 2019 and 2021, respectively, with 2,091 shared between them that were not present in 2023, again showing annotations being dropped (Venn diagram in Fig. 1c).

We saw similar patterns for RNA isoforms annotated as protein-coding, with 79,431 annotated in 2014 (Ensembl v76) and 89,374 in 2023 (Ensembl v109; 9,943 increase; Fig. 1d). Comparing protein-coding RNA isoforms between 2019, 2021, and 2023, 1,712 and 41 were unique to 2019 and 2021, respectively with 2,248 shared between them that were not present in 2023 (Venn diagram in Fig. 1d). Interestingly, there were 5 RNA isoforms annotated as protein-coding that were shared between 2019 and 2023 but were not annotated as protein-coding in 2021 (Venn diagram in Fig. 1d; Supplemental Table S2).

We also characterized the transcript biotype for all RNA isoforms in Ensembl v109 (2023; Fig. 1e). The top five annotation categories were protein-coding (with 89,374 RNA isoforms), lncRNA (56,103), retained intron (34,074), protein-coding CDS not defined (26,471), and nonsense-mediated decay (21,347; Fig. 1e).

#### RNA isoforms per gene

We quantified the distribution of RNA isoforms per gene body (i.e., all annotated regions where transcription occurs) in Ensembl v109 and, as expected, most gene bodies (38,690) have only one annotated isoform (median = 1; 75th percentile: 4; 85th : 8; 95th : 16; Fig. 1f). Comparatively, only 2,922 protein-coding genes (14.6%) have a single annotated isoform while the median number of annotated isoforms is 6 (75th percentile: 11 isoforms), demonstrating the transcriptional complexity among protein-coding genes compared to non-coding genes. Remarkably, the most annotated isoforms for a single gene body and a single protein-coding gene were 296 (*PCBP1-AS1*; ENSG00000179818; Fig. 1f) and 192 (*MAPK10*; ENSG00000109339; Fig. 1g), respectively. Similarly, we observed 7,255; 256; and 30 gene bodies with ≥ 10; ≥50; and ≥ 100 annotated isoforms, respectively, and 6,162; 169; and 94 protein-coding gene bodies for the same respective thresholds.

#### RNA isoform lengths and N50

Common read quality control metrics in RNA sequencing experiments include median read length and N50, which are generally used to assess RNA and sequencing quality. Thus, we wanted to quantify expected RNA lengths (median & N50) based on the annotations, as a reference; these values may not accurately represent expected RNA lengths in vivo, as this may change based on which gene bodies are being expressed and in what quantities, but it does provide a useful reference. As such, we quantified the median and N50 for all transcripts from 2014 to 2023. We also stratified transcripts by biotype and report the top three biotypes (based on 2023 annotations). Biotypes have changed over the years, including how long non-coding RNAs (lncRNA) are defined. Specifically, the lncRNA biotype was not present in Ensembl annotations before 2020 when the long intergenic non-coding RNA (lincRNA) biotype existed. Thus, for simplicity, we combined lncRNA and lincRNA biotypes for these purposes since current documentation lists lincRNAs as a subset of lncRNAs [8].

**Fig. 2:**
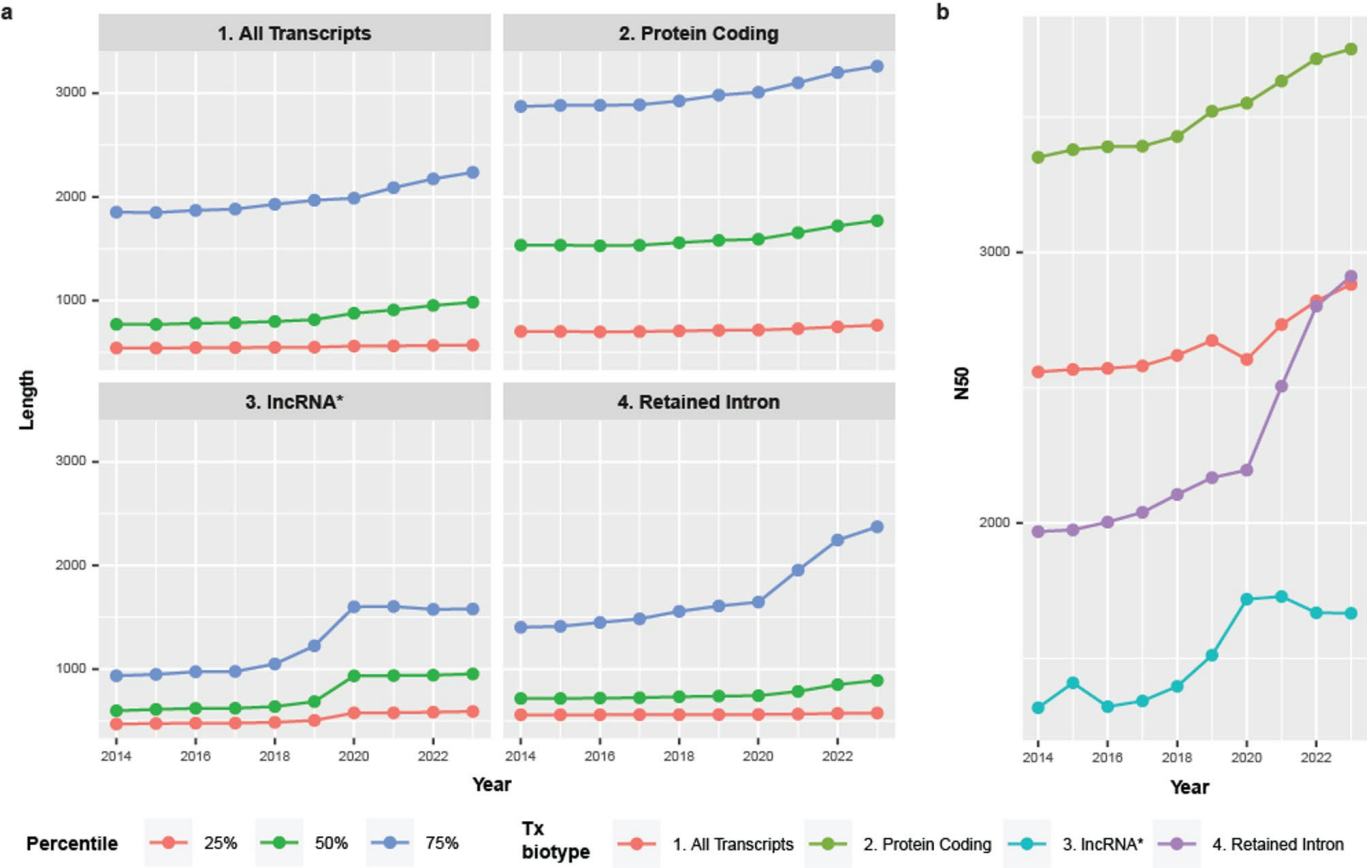
*Annotations reveal median and N50 transcript lengths across the years by biotype.***a** The 25th, 50th (median), and 75th percentiles of the aggregated transcripts (All Transcripts) as well as for the top three most abundant transcript biotypes in 2023 and how their values changed in the years leading up to 2023. The protein-coding biotype has the longest lengths for all three percentiles. **b** The N50 for each of the categories (All Transcripts, Protein Coding, lncRNA*, and Retained Intron). Again, Protein Coding transcripts boast the longest N50 values, ranging from 3,352 bp to 3,752 bp. The N50s generally increase across the years for all categories. (*) lncRNA is not a transcript biotype present in these Ensembl annotations before 2020, and lincRNA is only present in the annotations before 2020. Current documentation places lincRNA within the category of lncRNA, and as lincRNA and lncRNA are mutually exclusive in the annotations, we combined the lincRNA & lncRNA biotypes, for simplicity

The median read length across all transcripts ranged from 771 (2014) to 984 bp (2023; Fig. 2a, green line), with the 25th (red line) and 75th percentiles (blue line) ranging from 543 to 571 and 1,853 to 2,237, respectively. The median read length for protein-coding transcripts ranged from 1,535 bp in 2014 to 1,771 bp in 2023 and was > 500 bp longer than the other biotypes across all years. The median read lengths for lncRNA and retained intron biotypes are < 1 kb. There is a large increase in the lncRNA values from 2019 to 2020 (686 to 934, respectively), but this could be due to annotation nuances in definition between lincRNA and lncRNA. Looking at the N50s, the values for all transcripts in aggregate ranged from 2,559 bp to 2,882 bp. The protein-coding biotype has the largest N50s ranging from 3,352 bp to 3,752 bp, followed by retained intron (1,969 bp to 2,912 bp), and last, lncRNA (1,318 bp to 1,667 bp; Fig. 2b).

### New RNA isoforms between 2019 and 2023, including isoforms from medically relevant genes

**Fig. 3:**
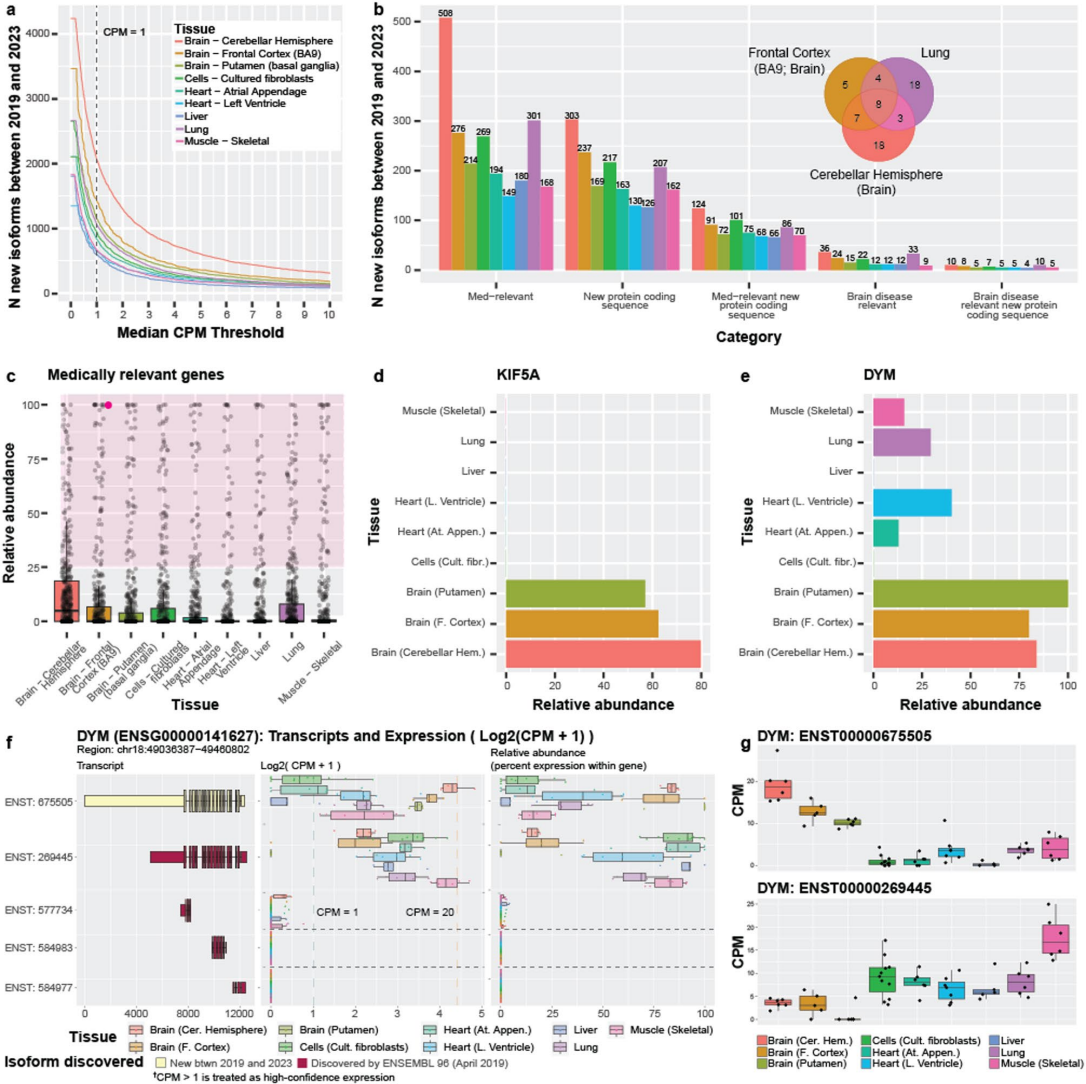
*The need to look deeper: Patterns across tissues for isoforms discovered between 2019 and 2023.***a** Isoforms discovered between 2019 and 2023 expressed across counts-per-million (CPM) thresholds. Expression above our standard thresholds (median unique counts ≥ 1 and CPM > 1) ranged from 594 (liver) to 2,054 (cerebellar hemisphere). (**b**-**e**) assume our standard thresholds. **b** Number of expressed isoforms discovered between 2019 and 2023 per tissue by categories. Venn shows isoform (new between 2019 and 2023) overlap for brain-disease-relevant genes in three tissues. **c** Relative abundance of isoforms from 497 medically relevant genes where ≥ 1 isoforms were annotated between 2019 and 2023, and expressed in at least one tissue. Each point represents the summed relative abundance for all new isoforms annotated between 2019 and 2023 for a given gene. The magenta point represents a gene where isoforms annotated between 2019 and 2023 constituted 100% of its expression in frontal cortex. The shaded area highlights genes where newly discovered isoforms constitute > 25% of its total expression for the given tissue. 171 (34.4%) of the genes had a combined relative abundance > 25% in all nine tissues, and 50 (10.1%) exhibited > 75% of relative abundance in ≥ 1 tissue. All 497 genes are plotted for each tissue. **d** Summed relative abundance of isoforms new between 2019 and 2023 for *KIF5A*, a brain-disease-relevant gene. Newly discovered isoforms constitute a major proportion of total expression for the gene. **e** Same as **d**, but for *DYM* (ENST00000675505), a medically relevant gene. (**f**-**g)** Plots from our R-shiny app. **f** Five isoforms from *DYM*, showing a cartoon of their exon structure (colored by discovery), the log2(CPM + 1) of the isoforms, and the relative abundance for each isoform. The first isoform, discovered between 2019 and 2023, is the most highly expressed isoform in several tissues. **g** Divergent expression patterns for the top *DYM* isoforms suggest preferential expression between different tissue groups (brain regions vs. the rest)

Many RNA isoforms were discovered between April 2019 (Ensembl v96) and February 2023 (Ensembl v109; 44,271), where 19,291 were newly annotated between 2019 and 2020 alone (Fig. 1c). To assess the value added in those four years of RNA isoform discovery, we quantified the expression of new isoforms within nine GTEx tissues (58 samples from GTEx long-read RNA-seq data sequenced by Glinos et al. [4]; Supplemental Table S3), using a threshold of median unique counts > 1 and counts per million (CPM) ≥ 1. The number of RNA isoforms discovered between 2019 and 2023 expressed in individual tissues ranged from 594 (liver) to 2,054 (cerebellar hemisphere; Fig. 3a), including 180 and 508 from medically relevant genes (Fig. 3b), respectively. Of the total new isoforms between 2019 and 2023, 126 and 303 isoforms contained new protein-coding sequence in liver and cerebellar hemisphere, respectively, and 66 and 124 were both medically relevant and had new protein-coding sequence (Fig. 3b).

Of the 2,054 new RNA isoforms discovered between 2019 and 2023 that are expressed in the cerebellar hemisphere, 36 are brain-disease-relevant, including notable genes such as *MAPT*, *SNCA*, and *KIF5A* (Fig. 3b). Exactly 33 brain-disease-relevant genes are also expressed in lung, which provides an example of disease-relevant genes that may have important functions beyond what they are most associated with. However, most of the isoforms from the brain-disease-relevant genes expressed in cerebellar hemisphere and lung do not overlap (Venn diagram in Fig. 3b). Similarly, 194 of the 829 RNA isoforms discovered between 2019 and 2023 expressed in atrial appendage heart tissue come from medically relevant genes, including *MED12*, *TPM1*, and *DYM*. For lung, 301 of 1053 new isoforms between 2019 and 2023 come from medically relevant genes.

Quantifying all RNA isoform expression patterns for a given gene across tissues is important to understanding its complexity and function, but arguably the “most important” isoforms may be the most highly expressed. Thus, we assessed relative abundance for isoforms annotated between 2019 and 2023 (compared to isoforms already known) in genes where ≥ 1 isoform was discovered in that period. Limiting to medically relevant gene bodies, we summed the relative abundance for all new isoforms between 2019 and 2023 for each of the 497 genes meeting these criteria. Of these, the isoforms discovered for 171 (34.4%) genes had a combined relative abundance > 25% in all nine tissues, showing that many of the isoforms discovered in that time consistently constituted a meaningful proportion of the gene’s expression; this result highlights the importance of their respective discoveries. Similarly, 50 (10.1%) of the 497 medically relevant genes with isoforms discovered between 2019 and 2023 constituted > 75% relative abundance (Fig. 3c). For the 452 genes with isoforms containing new protein-coding sequence discovered between 2019 and 2023, the combined relative expression of the new isoforms for 184 (40.7%) of the genes constituted > 25% relative abundance in all nine tissues. Total expression for the new isoforms for 72 (15.9%) of those genes constituted a relative abundance greater than 75% in at least one tissue. (Supplemental Figure S1; Supplemental Table S4)

Highlighting some genes for further study, *KIF5A* is a brain-disease-relevant gene with a large amount of its relative abundance stemming from isoforms discovered between 2019 and 2023, where the four new isoforms comprise 62.4%, 57.0%, and 79.8% of total gene expression for frontal cortex, putamen, and cerebellar hemisphere, respectively (Fig. 3d). *KIF5A* is implicated in several diseases, including spastic paraplegia 10 [9–11], neonatal intractable myoclonus [12, 13], and amyotrophic lateral sclerosis (ALS) [14–16]. For those with a vested interest in understanding and treating these diseases, knowing about and understanding all of *KIF5A*’s isoforms and their function is essential—perhaps especially in the context of interpreting the functional consequences of genetic variants [3].

*DYM* is another medically relevant gene, where mutations are known to cause Dyggve-Melchior-Clausen syndrome (DMC), a type of skeletal dysplasia also known to be associated with brain developmental defects [17, 18]. *DYM* was first named in 2003 and has 20 annotated isoforms in Ensembl v109. The overall median gene expression falls between approximately five and 25 CPM for the nine GTEx tissues included here (Supplemental Figure S2). Only two isoforms are expressed above our thresholds where one (ENST00000675505) is new between 2019 and 2023, has a new protein-coding sequence, and accounts for most of the gene expression in all three brain regions (frontal cortex: 80.0%; cerebellar hemisphere: 83.9%; putamen: 100.0%; Fig. 3e,f,g); this isoform is also present in four other tissues (skeletal muscle, lung, left ventricle [heart] and atrial appendage [heart]), but is not the primary isoform expressed. This described expression pattern may imply that these isoforms have tissue-specific roles.

## Discussion

Gene annotation is a monumental task, and we are grateful for large consortia and projects that compile, validate, and freely provide these invaluable resources to the scientific community. With the help of long-read sequencing technologies and approaches, the number of annotation entries has steadily increased [19–22], and will likely continue to increase, especially as sequencing technologies and analytical methods advance. Our analysis highlights that Ensembl updates introduce not only new genes, but new isoform annotations as well. While approximately 20,000 genes are annotated as protein-coding, the number of distinct protein-coding transcripts is far greater. Over the past decade, the continual refinement and validation of gene annotations have helped researchers better define gene locations, complexity, and function. Understanding how annotations evolve over time—and how rapidly they change—is crucial for interpreting research findings.

Many factors can influence the discovery of new isoforms. The populations and tissues used for isoform discovery will inevitably bias what isoforms can be discovered. Also, as a focus of gene and isoform discovery is to gain more insight into human health and disease, medically relevant genes are often deeply studied compared to other genes, resulting in a streetlamp effect where we identify a large proportion of new isoforms from those genes. Additionally, long-read sequencing has revolutionized isoform discovery and quantification, allowing for previously unidentifiable isoforms to be identified. As more long-read sequencing is performed, we anticipate isoform diversity will continue to grow. These factors are important to consider as we continue to study the transcriptome and identify the gaps present and how we will overcome them.

We highlighted that the number gene annotations varied across the years. Exactly why gene body annotations were dropped in subsequent releases is not clear, nor is it available in publicly accessible data (to our knowledge), but we point out the seeming inconsistencies to highlight how challenging annotating a genome is. Ensembl provides an ID History Converter tool that reports specific release versions whenever a gene ID has a major update. While useful for specific situations, the tool does not explicitly state what changed, nor could we access ID history via a high-throughput method (i.e., it required manual curation). Our analysis does not account for gene ID changes because, to our knowledge, extracting this information through Ensembl is not currently possible via a high-throughput method. Documentation on why changes are made and the ability to programmatically assess them genome-wide would increase transparency and improve scientific reproducibility. To be clear, however, Ensembl and their collaborators employ a manually supervised computational workflow combined with expert annotators to resolve these challenging problems [23], and their contributions to the field are both significant and essential.

In addition to quantifying the number of isoforms present in each annotation, we reported the median lengths and N50s across all isoforms. We showed that median length and N50 for all transcripts in aggregate maxes out in 2023 at 984 bp and 2,882 bp, respectively, while those for only protein-coding transcripts max out at 1,771 bp and 3,752 bp. Median read lengths and N50s are customarily used to assess RNA and sequencing quality, and assessing these metrics based on annotations estimates what read lengths would be necessary to fully sequence all isoforms from end to end. More experimental work is needed to determine whether these values represent reality in human RNA.

The continuous discovery of gene isoforms has significant implications. For example, in this study we highlighted a previously unknown *DYM* isoform, first annotated in 2023, that was the most highly expressed isoform of this gene in the brain. This underscores the importance of using the latest annotations—what was considered the predominant isoform yesterday may no longer hold that status today, particularly regarding which tissue type you investigate. Additionally, the recent Ensembl v114 annotation (May 2025) [24] reports 387,954 isoform annotations, roughly a 1.5 fold increase over the Ensembl v109 annotation (252,798 isoforms) we use in our analyses here. Using older annotations may lead researchers to “rediscover” isoforms already recognized in more recent updates or cause them to miss potentially important, disease relevant isoforms. Thus, the annotation version used matters, and to mitigate this issue, researchers should use the most up-to-date annotation when starting their analyses, while also reviewing previous (and newer, if applicable) versions when describing novel isoforms.

We argue that for RNA isoforms, the value of a reference genome is only as strong as its annotation, making high-quality annotations essential. But high-quality annotations are only the first step. In interrogating the Ensembl v109 annotation, we saw 7,255 genes with ≥ 10 annotated isoforms. This raises several important questions, including why genes need so many isoforms, and how many isoforms from a single gene are expressed at the same time. Additionally, it is unclear what roles isoforms play across tissues, and whether they function within the same pathways, or if their roles diverge. It is also unclear how they regulated. We examined two medically relevant genes, *KIF5A* and *DYM*. The distinct expression patterns of these genes suggest they may serve multiple functions. If this trend extends across the broader set of protein-coding genes, the differential expression of isoforms could provide critical insights into distinguishing disease states from normal tissue. In our view, this represents the future of personalized medicine and could inform new therapeutic strategies. Medically relevant isoforms may operate through distinct pathways depending on the tissue in which they are expressed. Furthermore, if they are regulated epigenetically or via splice variants, future studies should determine whether these regulatory mechanisms influence their expression and function differently. Investigating these questions across differentially expressed isoforms will be critical for identifying tissue- and disease-specific targets for pharmaceutical interventions.

Annotations are essential because they allow researchers to make connections between phenotypes and expression that we hope will result in actionable advances in our understanding of human health and disease. However, simply quantifying isoforms is not enough, we need to investigate the patterns. We need to understand whether isoforms expressed in different tissues are performing the same function, or even if they are expressed in the same tissue, whether the isoforms from the same gene perform the same function.

## Methods

### Downloading and comparing ensembl annotations

Gene transfer format (GTF) files for ten representative Ensembl GRCh38 annotations spanning ten years (2014–2023; one per year) were downloaded from the Ensembl website (Supplemental Table S1). Using Python version 3.11.3 within a Jupyter notebook (version 6.5.4) [25] (CODE AVAILABILITY), we quantified the number of genes and isoforms per year, as well as the number of protein-coding genes based on annotations within the GTF file. Specifically, for a gene or transcript (i.e., isoform) to be considered protein-coding, their respective “gene_biotype” and “transcript_biotype” must have been “protein_coding”. In all other instances, we use all genes (sometimes referred to herein as gene bodies) and isoforms annotated, regardless of their biotype. We performed set comparisons between the years 2019, 2021, and 2023 to identify the overlap between annotations. We used the Ensembl v109 (2023) annotations to quantify the most common transcript biotypes, as shown in Fig. 1e. To calculate the number of annotated isoforms per gene and per protein-coding gene (shown in Fig. 1f,g), respectively, we summed the number of unique transcripts (based on Ensembl transcript ID) for each unique Ensembl Gene ID. We also calculated the percentiles for annotated isoforms per gene using R version 4.3.1. The annotations from 2014 and 2015 had additional alternative contigs that we excluded from these analyses.

We assessed median transcript length and N50. We calculated the lengths of each transcript by summing the lengths of the exons that make up that transcript. We then determined the median read length and N50 for all transcripts for each annotation year. We also separated transcripts by transcript biotype and calculated median read length and N50 for each category.

### Downloading GTEx data, read pre-processing, genomic alignment, quality control, and analyses

We downloaded the GTEx nanopore long-read cDNA RNA-seq data (publicly available) from Glinos et al. [4] for this study from the AnVIL portal [26]. The data contains 88 GTEx samples across 15 different human tissues and cell-lines. We reprocessed the data using the methods used in our recent paper by Aguzzoli-Heberle et al. [3]. Sample pre-processing, alignment, and quality control were the same as Page et al. [27]. Briefly, the cDNA data was pre-processed using Pychopper (version 2.7.6), which identifies reads with the appropriate primers on both ends, rescues reads that have been fused together, orients them according to their genomic strand, and removes adapter/primer sequences. We applied the settings for the PCS109 sequencing kit, which was used on the GTEx data. Minimap2 [28] (version 2.26-r1175) was then used to align the pre-processed reads to GRCh38 without alternate contigs using the “ -x splice ” parameter suitable for spliced alignments (parameter settings: -k15 -w5 --splice -g2k -G200k -A1 -B2 -O2, 32 -E1, 0 -b0 -C9 -z200 -ub --junc-bonus =9 --cap-sw-mem=0 --splice-flank= yes) and the “ -uf ” parameter for identifying splicing sites using transcript strand. Samtools (version 1.17) was used to filter (requiring a Mapping Quality (MAPQ) score of 10 or greater), sort (by genomic coordinates), and index the BAM files. The BAM files were used as input to Bambu, which quantified the isoforms. The information on how to access all our scripts can be found in Code Availability. Quality control data for these samples is found in the original Glinos et al. [4] publication. See Page et al. [27] for the number of reads we analyzed with Bambu for this data, as this differs slightly from Glinos et al. due to minor differences in our pipelines.

#### Included Samples

Originally, 88 cDNA GTEx samples were sequenced by Glinos et al. Here, we use the same 58 samples across nine tissues in our analyses that were selected in Page et al. [27]. See that paper for inclusion criteria. Supplemental Table S3 lists all the included samples.

#### Expression of isoforms new between 2019 and 2023

We used the same counts matrices that were used in Page et al. [27]. The isoforms were filtered based on median unique counts > 1 and CPM > = 1 (by tissue). To assess the isoforms that were new between the Ensembl v96 annotation (released April 2019) and the Ensembl v109 annotation (released February 2023), we retained all Ensembl transcript ID’s from Ensembl v109 that were not present in v96. To quantify the percent of total expression for new isoforms since 2019 (as shown in Fig. 3c), we summed the relative abundance for those isoforms for a given gene.

### Figures and tables

We created figures and tables using a variety of python (version 3.11.3) and R (version 4.3.1) scripts. Some figures were downloaded from our Rshiny app (R version 4.3.0). All scripts are available on GitHub (see code availability). Isoform structures were visualized using R package ggtranscript [29] (version 0.99.9). Final figures were assembled using Adobe Illustrator.

## Supplementary Information

Supplementary Material 1

Supplementary Material 2

## Data Availability

GTEx long-read RNAseq data used is available through the AnVIL project26 at the following link: https://anvil.terra.bio/#workspaces/anvil-datastorage/AnVIL_GTEx_V9_hg38. Counts matrices from Bambu generated in this study can be downloaded here: https://doi.org/10.5281/zenodo.17092739. Data can be visualized on the Ebbert Lab website at https:/ebbertlab.com/gtex_rna_isoform_seq.html.

## Code availability

All code used in the manuscript is available at: https://github.com/UK-SBCoA-EbbertLab/ensembl_annotation_comparison.

## References

[1] Zhao S & Zhang B. A comprehensive evaluation of ensembl, RefSeq, and UCSC annotations in the context of RNA-seq read mapping and gene quantification. BMC Genomics. 2015;16:97. 10.1186/s12864-015-1308-8. 25765860 10.1186/s12864-015-1308-8 PMC4339237

[2] McCarthy DJ, et al. Choice of transcripts and software has a large effect on variant annotation. Genome Med. 2014;6:26. 10.1186/gm543. 24944579 10.1186/gm543 PMC4062061

[3] Aguzzoli Heberle B, et al. Mapping medically relevant RNA isoform diversity in the aged human frontal cortex with deep long-read RNA-seq. Nat Biotechnol. 2024;1–12.10.1038/s41587-024-02245-9. 38778214 10.1038/s41587-024-02245-9 PMC11863200

[4] Glinos DA, et al. Transcriptome variation in human tissues revealed by long-read sequencing. Nature. 2022;608:353–9. 10.1038/s41586-022-05035-y. 35922509 10.1038/s41586-022-05035-y PMC10337767

[5] Leung SK, et al. Full-length transcript sequencing of human and mouse cerebral cortex identifies widespread isoform diversity and alternative splicing. Cell Rep. 2021;37:110022. 10.1016/j.celrep.2021.110022 34788620 10.1016/j.celrep.2021.110022 PMC8609283

[6] Gene, CARD16 (ENSG00000204397.) - Summary - Homo_sapiens - Ensembl genome browser 103. http://feb2021.archive.ensembl.org/Homo_sapiens/Gene/Summary?db=core;g=ENSG00000204397;r=11:105041326-105101431;tl=XzM11mmNb0fURcoB-9564622.

[7] Gene, CARD16 (ENSG00000204397.) - Summary - Homo_sapiens - Ensembl genome browser 105. http://dec2021.archive.ensembl.org/Homo_sapiens/Gene/Summary?db=core;g=ENSG00000204397;r=11:105041326-105101431;tl=XzM11mmNb0fURcoB-9564622.

[8] Biotypes. https://useast.ensembl.org/info/genome/genebuild/biotypes.html

[9] Qiu Y, et al. A novel KIF5A gene variant causes spastic paraplegia and cerebellar ataxia. Ann Clin Transl Neurol. 2018;5:1415–20. 10.1002/acn3.650. 30480035 10.1002/acn3.650 PMC6243379

[10] Crimella C, et al. Mutations in the motor and stalk domains of KIF5A in spastic paraplegia type 10 and in axonal Charcot–Marie–Tooth type 2. Clin Genet. 2012;82:157–64. 10.1111/j.1399-0004.2011.01717.x 21623771 10.1111/j.1399-0004.2011.01717.x

[11] Goizet C, et al. Complicated forms of autosomal dominant hereditary spastic paraplegia are frequent in SPG10. Hum Mutat. 2009;30:E376–85. 10.1002/humu.20920. 18853458 10.1002/humu.20920

[12] Duis J, et al. KIF5A mutations cause an infantile onset phenotype including severe myoclonus with evidence of mitochondrial dysfunction. Ann Neurol. 2016;80:633–7. 10.1002/ana.24744 27463701 10.1002/ana.24744 PMC5042851

[13] Rydzanicz M, et al. KIF5A de Novo mutation associated with myoclonic seizures and neonatal onset progressive leukoencephalopathy. Clin Genet. 2017;91:769–73. 10.1111/cge.12831. 27414745 10.1111/cge.12831

[14] Brenner D, et al. Hot-spot KIF5A mutations cause Familial ALS. Brain. 2018;141:688–97. 10.1093/brain/awx370. 29342275 10.1093/brain/awx370 PMC5837483

[15] Nicolas A, et al. Genome-wide analyses identify KIF5A as a Novel ALS gene. Neuron. 2018;97:1268–e12836. 10.1016/j.neuron.2018.02.027. 10.1016/j.neuron.2018.02.027 PMC5867896 29566793

[16] Pant DC, et al. ALS-linked KIF5A ∆Exon27 mutant causes neuronal toxicity through gain‐of‐function. EMBO Rep. 2022;23:e54234. 10.15252/embr.202154234. 35735139 10.15252/embr.202154234 PMC9346498

[17] El Ghouzzi V, et al. Mutations in a novel gene Dymeclin (FLJ20071) are responsible for Dyggve–Melchior–Clausen syndrome. Hum Mol Genet. 2003;12:357–64.10.1093/hmg/ddg029.> 12554689 10.1093/hmg/ddg029

[18] Gaboon NEA, et al. A Novel Homozygous Frameshift Variant in DYM Causing Dyggve-Melchior-Clausen syndrome in Pakistani patients. Front Pediatr. 2020;8:383. 10.3389/fped.2020.00383. 32766185 10.3389/fped.2020.00383 PMC7378890

[19] Dyer SC, et al. Ensembl 2025. Nucleic Acids Res. 2025;53:D948–57. 10.1093/nar/gkae1071. 39656687 10.1093/nar/gkae1071 PMC11701638

[20] Frankish A, et al. GENCODE reference annotation for the human and mouse genomes. Nucleic Acids Res. 2019;47:D766–73. 10.1093/nar/gky955. 30357393 10.1093/nar/gky955 PMC6323946

[21] Mudge JM, et al. GENCODE 2025: reference gene annotation for human and mouse. Nucleic Acids Res. 2025;53:D966–75. 10.1093/nar/gkae1078. 39565199 10.1093/nar/gkae1078 PMC11701607

[22] Kaur G, et al. GENCODE: massively expanding the lncRNA catalog through capture long-read RNA sequencing. bioRxiv. 2024. 10.1101/2024.10.29.620654.39763835

[23] Aken BL, et al. The Ensembl gene annotation system. Database (Oxford). 2016. 10.1093/database/baw093. 27337980 10.1093/database/baw093 PMC4919035

[24] Ensembl. genome browser 114. http://May2025.archive.ensembl.org/index.html

[25] Project Jupyter. https://jupyter.org

[26] Schatz MC, et al. Inverting the model of genomics data sharing with the NHGRI genomic data science Analysis, Visualization, and informatics Lab-space. Cell Genomics. 2022;2:100085. 10.1016/j.xgen.2021.100085. 35199087 10.1016/j.xgen.2021.100085 PMC8863334

[27] Page ML et al. A bioinformatic survey of RNA isoform diversity and expression across 9 GTEx tissues using long-read sequencing data. BMC Genomics - Rev. 2024) 10.1186/s12864-025-11919-w. 10.1186/s12864-025-11919-w PMC12679790 41345545

[28] Li H. Minimap2: pairwise alignment for nucleotide sequences. Bioinformatics. 2018;34:3094–100. 10.1093/bioinformatics/bty191. 29750242 10.1093/bioinformatics/bty191 PMC6137996

[29] Gustavsson EK, Zhang D, Reynolds RH, Garcia-Ruiz S, Ryten M. ggtranscript: an R package for the visualization and interpretation of transcript isoforms using ggplot2. Bioinformatics. 2022;38:3844–3846. 10.1093/bioinformatics/btac409. 35751589 10.1093/bioinformatics/btac409 PMC9344834

