# Supplementary material for: Genome annotations matter: characterizing Ensembl hg38 annotations from 2014 to 2023
Source: BMC Genomics. 2025 Dec 5;26:1079. doi: 10.1186/s12864-025-12167-8 (PMC12679773; doi:10.1186/s12864-025-12167-8)
Supplement: Supplementary file 1 — Supplementary Material 1 [file 12864_2025_12167_MOESM1_ESM.pdf]

Supplementary Tables:

Supplementary Table 1: Ten Ensembl annotations (one per year) used as an overview from 2014-2023 and their download paths.

Supplementary Table 2: Gene and transcripts that were labeled as protein-coding in 2019 and 2023 but not in 2021

Supplementary Table 3: Included GTEx samples

Supplementary Table 4: Number of genes expressing isoforms new since 2019 at different relative abundance thresholds.

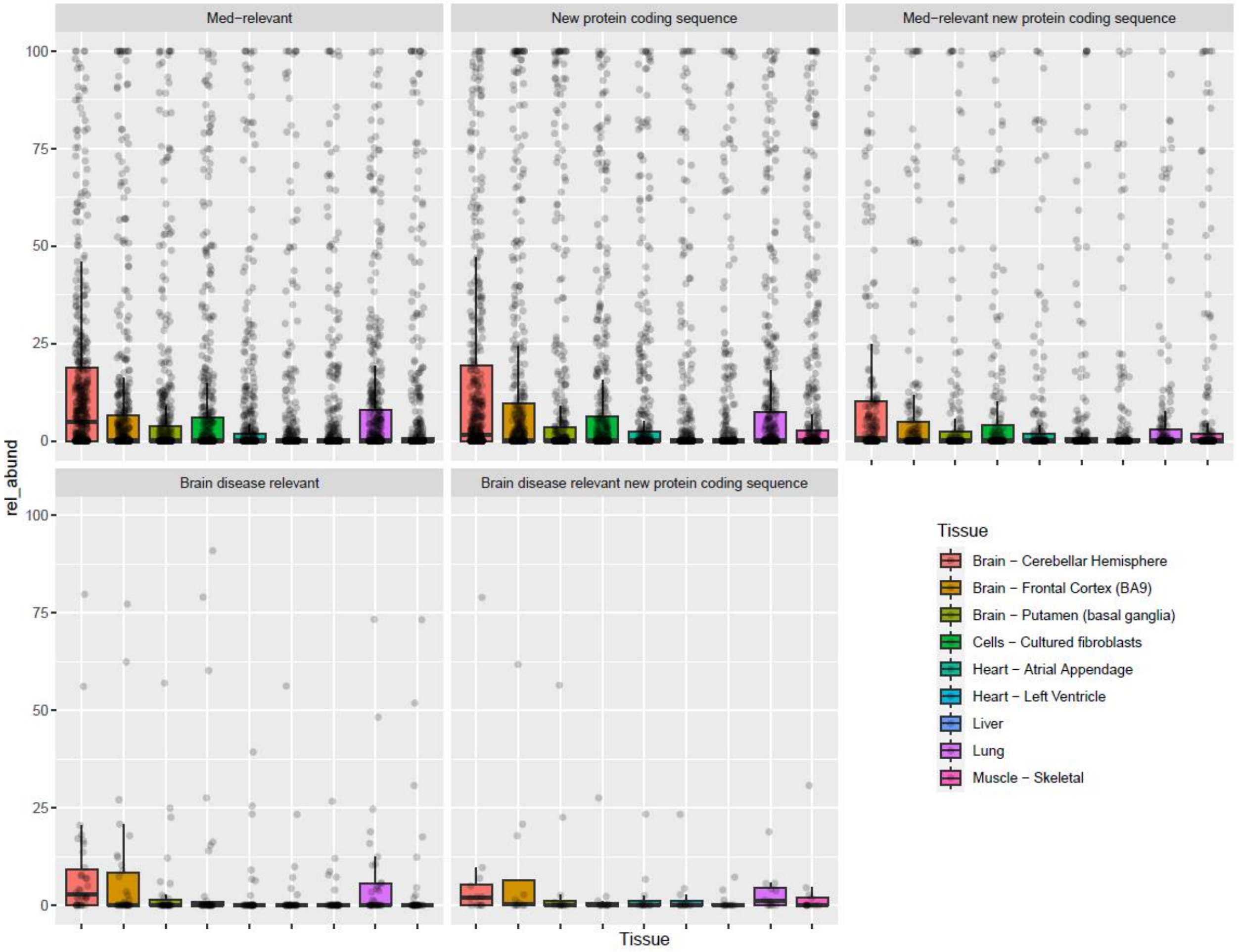

Supplemental Figure 1: Percent relative abundance across isoform categories

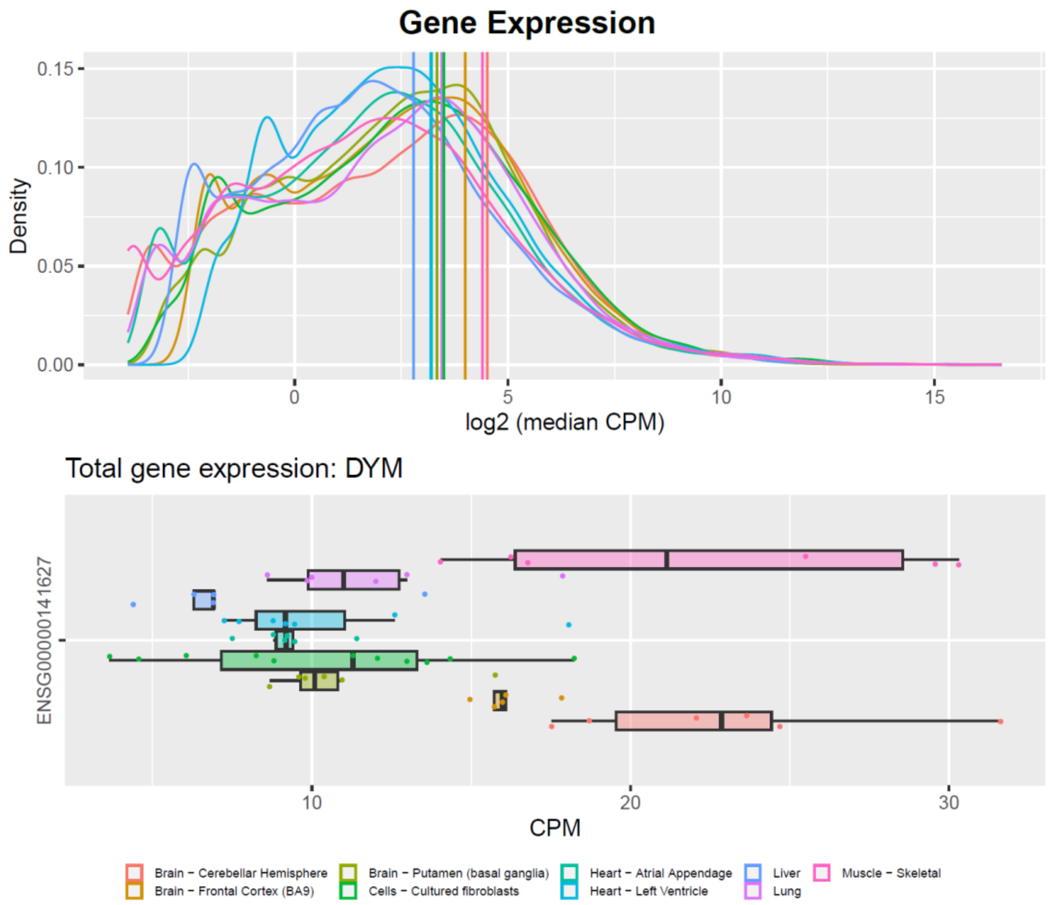

Supplemental Figure 2: Gene expression of DYM. Total gene expression of DYM across tissues. Density plot showing where the gene falls on the distribution of all genes expressed in each tissue.
